# Pre-procedural abnormal von Willebrand factor function predicts clinical outcomes after Transcatheter Aortic Valve Implantation: a prospective cohort study

**DOI:** 10.3389/fcvm.2025.1576921

**Published:** 2025-05-23

**Authors:** Haitham Abu Khadija, Mohammad Alnees, Omar Ayyad, Gera Gandelman, Nizar Abu Hamdeh, Amir Haim, Yazan Hamdan, Ramon Cohen, Duha Najajra, Alena Kirzhner, Tal Schiller, Jacob George, Alex Blatt

**Affiliations:** ^1^ Department of Cardiology, Kaplan Medical Center and Faculty of Medicine, Hebrew University of Jerusalem, Rehovot, Israel; ^2^ Postgraduate Medical Education, Global Clinical Scholer Research Training Program, Harvard Medical School, Boston, MA, United States; ^3^ Department of Internal Medicine B, Kaplan Medical Center and Faculty of Medicine, Hebrew University of Jerusalem, Rehovot, Israel; ^4^ Department of Internal Medicine A, Kaplan Medical Center and Faculty of Medicine, Hebrew University of Jerusalem, Rehovot, Israel; ^5^ Department of Diabetes, Endocrinology and Metabolism, Kaplan Medical Center and Faculty of Medicine, Hebrew University of Jerusalem, Rehovot, Israel

**Keywords:** transcatheter aortic valve implantation (TAVI), acquired von willebrand syndrome (AvWS), platelet count, major vascular complications, predictive modeling

## Abstract

**Background and objectives:**

Transcatheter Aortic Valve Implantation (TAVI) is a minimally invasive intervention for aortic stenosis, which is associated with the potential for major vascular complications and arrhythmias. This study aims to identify primary predictors of these complications, emphasizing the roles of Decreased Platelet Count (DPC) and Acquired Von Willebrand Syndrome (AVWS).

**Methods:**

We performed a prospective study with 80 patients planning to receive TAVI at the Heart Center, Kaplan Medical Center, Rehovot, Israel. Pre-procedural evaluations include the measurement of baseline platelet counts and the functionality of the von Willebrand factor. The DPC was determined as the percentage decreased from baseline to the lowest count. AVWS was diagnosed through the assessment of von Willebrand factor activity and antigen concentrations.

**Results:**

Our results demonstrate that both DPC and AVWS are crucial predictors of major vascular complications. Specifically, patients with a DPC exceeding 20% exhibited a coefficient (Coef) of 1.276 (*p* = 0.072; 95% CI: −0.116 to 2.668) for complications. While, patients with abnormal von Willebrand factor function presented an Coef of 1.841 (*p* = 0.022; 95% CI: 0.271–3.410) for complications compared to those without AVWS. ROC curve analysis indicated an AUC of 0.7417 for the DPC model and 0.8025 for the AVWS model in predicting major vascular complications. In the arrhythmia model, AVWS appeared as a significant predictor of arrhythmias, with an OR of 4.480 [95% CI: (1.21, 16.49), *p* = 0.024].

**Conclusions:**

Assessing both DPC and von Willebrand factor function is crucial for predicting post-TAVI complications.

## Introduction

Aortic stenosis (AS) is a prevalent valvular heart disease in the elderly, characterized by the narrowing of the aortic valve orifice. This obstruction increases afterload, leading to left ventricular hypertrophy and potentially heart failure. As the aging population continues to grow, the incidence of AS is expected to rise, necessitating effective treatment options (1). Transcatheter Aortic Valve Implantation (TAVI) has emerged as a minimally invasive alternative to surgical valve replacement, offering significant benefits for patients deemed moderate to high risk for traditional surgery (2).

Despite its advantages, TAVI is associated with various complications, including major vascular complications and arrhythmias, which can significantly affect patient outcomes (3–6). Recent studies have suggested that changes in platelet count post-TAVI may serve as important indicators of these complications (7, 8). Specifically, the percentage decrease in platelet count (DPC) during the nadir phase- the lowest recorded platelet counts during hospitalization- following the procedure has been correlated with adverse outcomes (9). Additionally, acquired von Willebrand syndrome (AVWS) has been recognized as a potential risk factor in patients undergoing TAVI, further complicating the clinical landscape (10, 11).

However, there is still limited research investigating the interplay between the decrease in platelet count (DPC), acquired von Willebrand syndrome (AVWS), and their combined effects on complications following TAVI. This study aims to fill this gap by identifying key predictors of major vascular complications and arrhythmias in patients undergoing TAVI through assessing the predictive value of pre-procedural abnormal von Willebrand Factor function in comparison to DPC after TAVI.

## Materials and methods

### Study design and population

This prospective study was conducted at the Heart Center, Kaplan Medical Center, Rehovot, Israel, enrolling patients with severe symptomatic aortic stenosis referred for transcatheter aortic valve implantation (TAVI) between January 2020 and December 2021.

Severe aortic stenosis is defined by one of the following criteria: an aortic valve area of less than 1.0 cm^2^, an indexed valve area of less than 0.6 cm^2^/m^2^, a mean gradient exceeding 40 mmHg, a maximum jet velocity greater than 4.0 m/s, or a velocity ratio of less than 0.25 (12). Eligibility for TAVI was determined by the local Heart Team after a thorough evaluation of each case.

The study was performed according to the policies of the Declaration of Helsinki, and authorized by the Institutional Ethics Committee, Kaplan Medical Center (confirmation 0091-20-KMC). Exclusion criteria included patients with chronic systemic inflammatory or autoimmune diseases, acute infections, hematological disorders, those whose von Willebrand factor (vWF) levels were not measured at baseline, Patients with a baseline platelets count <100 × 10^9^/L and those without follow-up blood tests. Patients who experienced periprocedural death within 72 h after TAVI were also excluded.

The SEV (self-expandable valve)-treated patients were implanted with the following valves: corevalve, evolute-R, or evolute-PRO (Medtronic, Inc., Minneapolis, Minnesota). While, BEV (ballon-expandable valve)- treated patients were implanted with the following valves: Sapien XT, or S3 (Edwards Lifesciences, Irvine, California). All interventions were performed via transfemoral access, the safety wire technique, along with the Prostar XL vascular closure device (Abbott Vascular, Redwood City, California) were used for the trans-femoral artery access and closure. We considered the procedure duration to be “skin to skin”. The start time was the arterial access opening, and the end time was when we closed this access. The procedures were conducted in hybrid operating rooms under either general anesthesia or local anesthesia with conscious sedation. It is important to note that transcatheter aortic valve-in-valve implantation was not part of this study. Informed consent was obtained from all participants, ensuring adherence to ethical standards throughout the research process.

### Sample size calculation

We conducted a sample size calculation using a two-sample means independent *t*-test to determine the necessary sample size for our study. Our primary focus was to evaluate how effectively the von Willebrand Factor (vWF) activity-to-antigen (vWF: Ac/vWF: Ag) ratio predicts the occurrence of major vascular complications. We based our parameter estimates on previous studies that reported mean vWF activity values and variances (13). We set a two-sided significance level (*α*) of 0.05 and aimed for a power of 0.80 to detect meaningful differences between the two groups. Specifically, the anticipated mean for the major bleeding group (m1) was 109, while the mean for the no major bleeding group (m2) was 164, with standard deviations of 31 and 99, respectively. Our calculations indicated that a total sample size of 60 participants would be necessary, resulting in 30 participants per group. This sample size ensures adequate power to detect differences in vWF: Ac/vWF: Ag related to the risk of the occurrence of major vascular complications following TAVI.

### Periprocedural antithrombotic regimens

For periprocedural pharmacological management, patients who were not on anticoagulants or antiplatelet therapy before TAVI were prescribed a daily dose of 75 mg of oral antiplatelet medication after the procedure. This regimen was continued for life and maintained during hospitalization unless a major bleeding event occurred. Patients on chronic oral anticoagulant (OAC) therapy discontinued their medication at least 48 h before the procedure and resumed OAC therapy post-TAVIFollowing ESC guidelines, single antiplatelet therapy (SAPT) with aspirin (100 mg) or clopidogrel (75 mg) was initiated the day before the procedure and continued for six months, unless patients required DAPT which is combination of aspirin and a P2Y12 inhibitor (e.g., clopidogrel) for patients with recent stent insertion. OAC was used as a single agent when indicated. During the procedure, unfractionated heparin (UFH) was administered to maintain an activated clotting time (ACT) greater than 300 s, with reversal achieved using protamine sulfate at a dosage of 1 gram per 100 units of heparin administered.

### Blood sampling and laboratory assays

For all study participants, venous peripheral blood samples were collected at two time points: the day before the procedure and the third day post-procedure. Blood samples were drawn into standardized collection tubes containing 3.2% trisodium citrate at room temperature and were centrifuged within 30 min according to the manufacturer's instructions (15 min at 1,500 × g). The resulting plasma was then frozen at −80°C and stored for a maximum of six months.

Von Willebrand Factor was assessed using the following parameters: (1) vWF activity (vWF: Ac; INNOVANCE® VWF Ac), (2) vWF antigen (vWF:Ag; VWF Ag®), and the activity-to-antigen ratio (vWF:Ac/vWF:Ag). Standard human plasma, sourced from Siemens Healthcare Diagnostics in Eschborn, Germany, was utilized for calibration. The measurements were conducted as part of routine laboratory analyses using the Siemens Behring Coagulation System XP®.

### Definitions and endpoints

According to Jiritano et al. from the PORTRAIT study (14). The average time for obtaining the nadir platelet count value was three days after implantation, and it was determined using this formula:

DPC=100×[baselineplateletcount–nadirplateletcount]/baselineplateletcount.

According to Ibrahim et al. (15), the nadir DPC cutoff of 20% was chosen for analysis. Regarding the von Willebrand Factor (vWF) activity-to-antigen (vWF: Ac/vWF: Ag) ratio before TAVI, several cutoff values have been documented in the literature, including 80% for vWF abnormalities (16) and 70% for acquired von Willebrand syndrome (17, 18). In this study, **a cutoff of 70% was selected** to assess potential causative factors associated with acquired von Willebrand syndrome.

Clinically suspected Heyde's syndrome was defined as the presence of severe aortic stenosis alongside a documented history of gastrointestinal angiodysplasia and gastrointestinal bleeding confirmed by endoscopy (19). To evaluate bleeding complications and arrhythmia, we applied the definitions established by the Valve Academic Research Consortium-3 (VARC-3) (20). In our study, we specifically targeted several key outcomes, including major vascular complications, major bleeding, acute kidney injury (AKI), myocardial infarction (MI), pacemaker insertion, re-admission, and arrhythmias. These endpoints were assessed to provide a thorough evaluation of patient outcomes following TAVI.

### Statistical analysis

**Figure 1:**
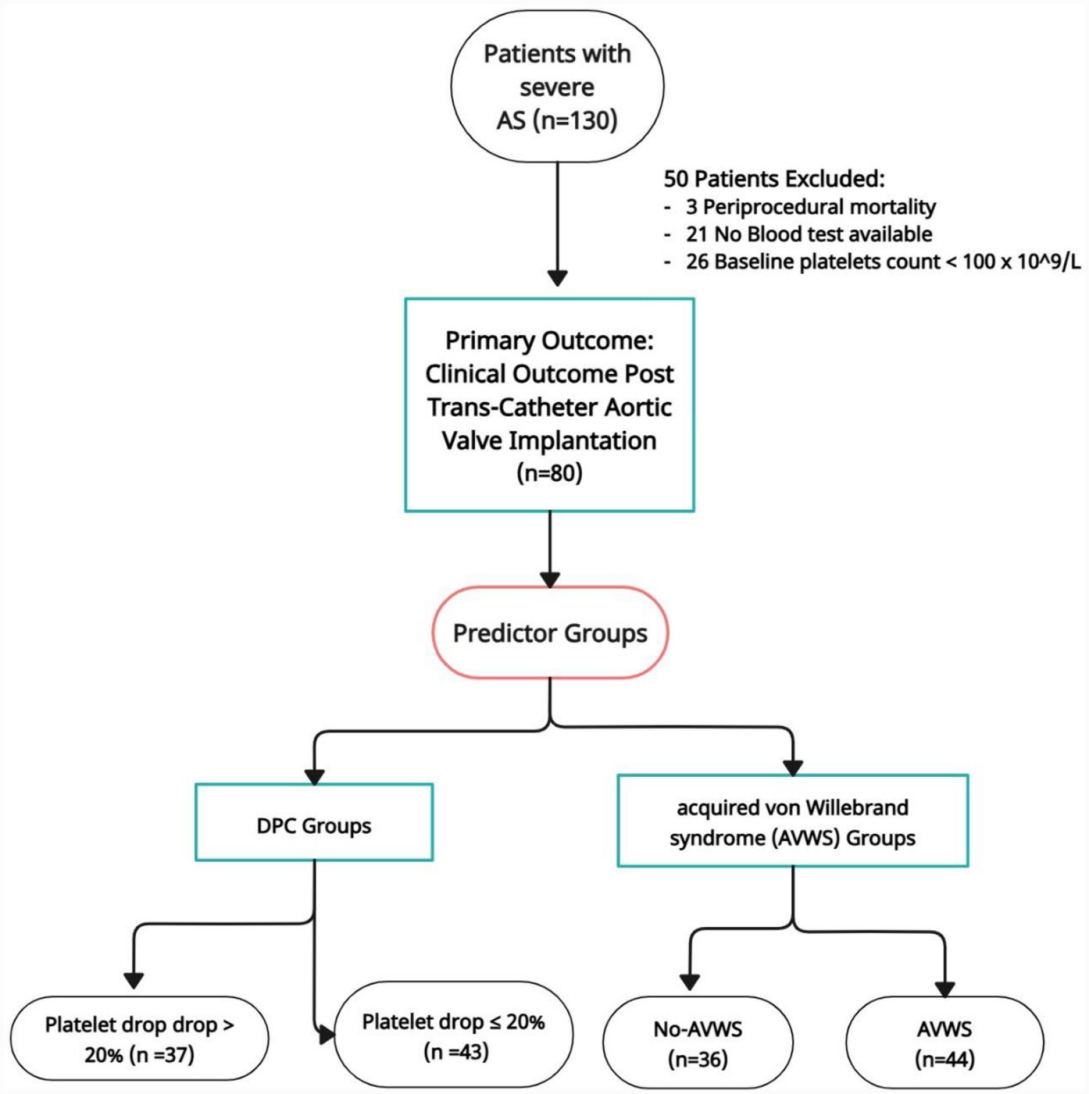
Flowchart patients.

Data were analyzed on a complete-case basis. Patients with missing values in key predictors or outcome variables—such as follow-up laboratory tests, von Willebrand factor measurements, procedural characteristics, or baseline thrombocytopenia—were excluded from the final multivariable analysis. Specifically, 21 patients were excluded due to missing follow-up lab results, 3 due to periprocedural death, and 26 due to baseline thrombocytopenia. Consequently, the final analysis was conducted on 80 patients with complete data. No imputation methods were applied (see Figure 1).

Continuous variables are presented as mean ± standard deviation and were compared using Student's *t*-test, Mann–Whitney *U* test, as appropriate. For categorical variables, which are expressed as counts and percentages, comparisons were made using the *χ*^2^ test or Fisher's exact test. All reported *p*-values are two-sided, with a significance threshold set at *p* < 0.05. Data analysis was conducted using STATA software, version 17.

### Construction and validation of the model

We utilized an inter-method approach to develop a logistic regression model. The model's performance was assessed using likelihood ratio chi-square tests, variance inflation factors, and the Hosmer-Lemeshow test. To investigate nonlinearity, we applied a Generalized Additive Model.

To examine the predictors of major vascular complications, we employed multivariable logistic regression models. Two models were developed: the DPC Model included predictors such as Decrease in Platelet Count (DPC), post-dilatation, procedural duration, left ventricular ejection fraction, septum thickness, and obesity. The AVWS Model included all predictors from the DPC model Except for a Decrease in Platelet Count (DPC) replaced by acquired von Willebrand syndrome (AVWS).

We included clinically relevant covariate obesity in our multivariable models, despite its lack of statistical significance in univariate analyses. This decision was informed by established evidence linking these metabolic risk factors to adverse cardiovascular outcomes and procedural complications, particularly in patients undergoing structural heart interventions (21–24). Given the inclusion of seven predictors and only thirteen outcome events, the resulting events-per-variable (EPV) ratio was below the conventionally accepted threshold of 10, raising concerns about model overfitting. To address this limitation, we employed Firth's penalized likelihood regression, a bias-reduction method designed to produce more reliable parameter estimates in small-sample and sparse-data settings. This approach mitigates the inflation of effect sizes and convergence issues commonly associated with traditional logistic regression under similar conditions, thereby enhancing the stability and credibility of our findings.The Akaike Information Criterion (AIC) and Bayesian Information Criterion (BIC) were calculated for both models to assess their relative fit. A lower AIC or BIC indicates a better fit while penalizing for the number of predictors used. The likelihood ratio test was initially considered for model comparison; however, due to both models having the same degrees of freedom, the models were determined to be non-nested, making the likelihood ratio test inappropriate.

To assess discriminative ability, we calculated the area under the receiver operating characteristic curve (AUC) and compared the two models using the DeLong method via the roccomp command in Stata version 17. Due to post-estimation limitations of the firthlogit command, we were unable to directly extract predicted probabilities from the Firth regression models. As a result, ROC curves and AUC values were derived using standard logistic regression models with identical covariates. While this approximation does not incorporate Firth's penalized likelihood correction, it provides a reasonable and interpretable comparison of the models' discriminative performance.

## Results

Table 1 presents the characteristics of patients undergoing Transcatheter Aortic Valve Implantation (TAVI) stratified by the degree of platelet count decrease (DPC) post-procedure. Patients are categorized into two groups: those with a platelet drop greater than 20% (DPC > 20%, *n* = 37) and those with a platelet drop of 20% or less (DPC ≤ 20%, *n* = 43). The table summarizes demographic data, medical history, procedural parameters, hemostatic parameters, and complications within 30 days post-TAVI. The mean age of patients with a DPC > 20% is 80.6 years, compared to 78.4 years for those with a DPC ≤ 20%, but this difference is not statistically significant (*p* = 0.275), suggesting age may not be a key factor in platelet drops. Gender distribution shows a higher percentage of males in the DPC ≤ 20% group, yet this finding is also not significant (*p* = 0.115). Similarly, body mass index (BMI) and STS scores do not show significant differences between groups (*p* = 0.692 and *p* = 0.526, respectively), indicating that obesity and surgical risk do not correlate with platelet changes. Chronic conditions such as diabetes, hypertension, and atrial fibrillation have similar prevalence rates in both groups, with no significant differences found for coronary artery disease or other medical histories, further supporting that these factors are unlikely to influence DPC. Regarding antiplatelet and anticoagulant use, there are no significant differences in therapy types, suggesting these do not significantly impact platelet counts post-TAVI. Echocardiographic parameters also show no significant differences, reinforcing the idea that cardiac function does not correlate with platelet changes. Notably, fewer patients in the DPC > 20% group underwent post-dilatation (*p* = 0.004), and the procedural duration was significantly longer in this group (*p* = 0.0190), indicating potential procedural variations for patients with greater platelet drops. At nadir, the platelet count was significantly lower in the DPC > 20% group (*p* = 0.0003), and thrombocytopenia was more prevalent (*p* = 0.010), suggesting that a significant drop in platelet count is associated with an increased risk of complications, particularly major vascular complications (*p* = 0.030). In summary, while many demographic and clinical factors do not appear to influence platelet count changes, procedural aspects and complications highlight significant associations, emphasizing the need for careful monitoring in patients experiencing substantial platelet decreases post-TAVI.

**Table 1:** Characteristics of patients and characteristics of trans-catheter aortic valve implantation (TAVI) according to decrease in platelet count (DPC) groups.

Variable	Category	Platelet drop > 20% (*n* = 37)	Platelet drop ≤ 20% (*n* = 43)	*P*-value
Demographic data				
Age, y	Mea*n* ± SD	80.621 ± 7.499	78.372 ± 10.325	0.275^a^
Gender *n*, %	Male	16 (20.00)	27 (33.75)	0.115^b^
	Female	21 (26.25)	16 (20.00)	
BMI, mean ± SD, kg/m^2^	Mean ± SD	28.269 ± 5.262	27.818 ± 4.865	0.692^a^
Obesity (BMI ≥ 30 kg/m^2^), *n* (%)	No	21 (26.25)	31 (38.75)	0.167^b^
	Yes	16 (20.00)	12 (15.00)	
STS Score	Mean ± SD	7.22 ± 1.22	6.98 ± 1.33	0.526^a^
Medical History				
Diabetes Mellitus	No	21 (26.25)	24 (30.00)	1.000^b^
	Yes	16 (20.00)	19 (23.75)	
Hypertension	No	8 (10.00)	3 (3.75)	0.101^b^
	Yes	29 (36.25)	40 (50.00)	
Smoker	No	30 (37.50)	33 (41.25)	0.785^b^
	Yes	7 (8.75)	10 (12.50)	
Dyslipidemia	No	3 (3.75)	10 (12.50)	0.078^b^
	Yes	34 (42.50)	33 (41.25)	
Atrial fibrillation	No	22 (27.50)	24 (30.00)	0.822^b^
	Yes	15 (18.75)	19 (23.75)	
Coronary Artery Disease (CAD)	No	15 (18.75)	17 (21.25)	1.00^b^
	Yes	22 (27.50)	26 (32.50)	
Peripheral Vascular Disease (PVD)	No	27 (33.75)	28 (35.00)	0.479^b^
	Yes	10 (12.50)	15 (18.75)	
S/PMyocardial Infarction	No	27 (33.75)	33 (41.25)	0.798^b^
	Yes	10 (12.50)	10 (12.50)	
S/PCerebrovascular Accident—Transient Ischemic Attack	No	33 (41.77)	35 (44.30)	0.528^b^
	Yes	4 (5.06)	7 (8.86)	
Pacemaker	No	28 (35.00)	33 (41.25)	1.00^b^
	Yes	9 (11.25)	10 (12.50)	
S/P CABG	No	33 (41.25)	38 (47.50)	1.00^b^
	Yes	4 (5.00)	5 (6.25)	
S/PTAVI PCI	No	24 (30.00)	25 (31.25)	0.647^b^
	Yes	13 (16.25)	18 (22.50)	
Antiplatelets				
SAPT	No	34 (42.50)	35 (43.75)	0.20^b^
	Yes	3 (3.75)	8 (10.00)	
DAPT	No	35 (43.75)	42 (52.50)	0.58^b^
	Yes	2 (2.50)	1 (1.25)	
Anticoagulants	No	33 (41.25)	37 (46.25)	0.74^b^
	Yes	4 (5.00)	6 (7.50)	
Preprocedural TTE				
LV eDD	Median (Q1–Q3)	46 (42–52)	45 (41–49)	0.397^c^
Septum, mean ± SD		12.540 ± 1.834	13.348 ± 2.213	0.082^a^
LVEF	Median (Q1–Q3)	55 (55–60)	55 (45–55)	0.085^c^
The posterior wall	Median (Q1–Q3)	11 (10–12)	12 (10–13)	0.149^c^
Mitral Regurgitation	None	2 (2.53)	0 (0.00)	**0.036^b^**
	Mild	23 (29.11)	22 (27.85)	
	Moderate	8 (10.13)	20 (25.32)	
	Severe	3 (3.80)	1 (1.27)	
Procedural parameters				
Valve size	Median (Q1–Q3)	27 (26–29)	26 (26–29)	0.99^c^
Post dilatation	No	22 (27.50)	38 (47.50)	**0.004^b^**
	Yes	15 (18.75)	5 (6.25)	
Valve types	SEV	26 (32.50)	22 (27.50)	0.10^b^
	BEV	11 (14.10)	21 (26.92)	
Procedural duration (m)	Median (Q1–Q3)	74 (66–88)	63 (55–86)	**0.019^c^**
Contrast medium volume (ml)	Median (Q1–Q3)	93 (70–140)	100 (70–125)	0.794^a^
Valve Type	SEV	25 (32.05)	21 (26.92)	0.107^b^
	BEV	11 (14.10)	21 (26.92)	
x-ray exposure value (mGy·cm^2^)	Median (Q1–Q3)	37,636 (22,211- 64,458)	37,123 (21,951- 77,692)	0.66^c^
Hemostatic parameters				
Baseline				
Platelet count,10³/µl	Median (Q1–Q3)	187 (147–219)	162 (135–198)	0.235^c^
vWF:Ac/Ag ratio (%)	Median (Q1–Q3)	0.56 (0.41–0.87)	0.75 (0.45-.88)	0.275^c^
Acquired von Willebrand syndrome (AVWS), *n*	No	13 (16.25)	23 (28.75)	0.11^b^
	Yes	24 (30.00)	20 (25.00)	
Prothrombin time, seconds	Median (Q1–Q3), *n* = 68	55 (11.5–88)	67 (11.2–86)	0.829^c^
Activated partial thromboplastin time, seconds	Median (Q1–Q3), *n* = 68	25.4 (24–27.7)	25.8 (24.5–27.8)	0.494^c^
International Normalized Ratio (INR)	Median (Q1–Q3), *n* = 68	1.09 (1.01–1.17)	1.06 (1.01–1.12)	0.388^c^
At nadir (3 days post-procedure)				
Platelet count,10³/µl	Median (Q1–Q3)	101 (85–146)	152 (117–179)	**<0.001^c^**
Thrombocytopenia, *n* (%)	No	8 (10.00)	22 (27.50)	**0.010^b^**
	Yes	29 (36.25)	21 (26.25)	
Prothrombin time, seconds	Median (Q1–Q3), *n* = 18	45 (13.1–84)	76 (48–98)	0.29^c^
Activated partial thromboplastin time, seconds	Median (Q1–Q3), *n* = 18	27 (23.3–28.8)	25.2 (24.7–39.5)	0.61^c^
International Normalized Ratio (INR)	Median (Q1–Q3), *n* = 18	1.12 (1.02–1.49)	1.06 (0.99–1.2)	0.388^c^
Complications up to 30 days post-TAVI				
AKI	No	30 (37.50)	40 (50.00)	0.174^b^
	Yes	7 (8.75)	3 (3.75)	
MI	No	36 (45.00)	41 (51.25)	1.00^b^
	Yes	1 (1.25)	2 (2.50)	
Major Bleeding	No	35 (43.75)	42 (53.16)	0.59^b^
	Yes	2 (2.50)	1 (1.27)	
Pacemaker	No	34 (42.50)	39 (48.75)	1.00^b^
	Yes	3 (3.75)	4 (5.00)	
Re-admission	No	32 (40.00)	37 (46.25)	1.00^b^
	Yes	5 (6.25)	6 (7.50)	
Arrhythmias	No	26 (32.50)	31 (38.75)	1.00^b^
	Yes	11 (13.75)	12 (15.00)	
Major vascular complications	No	27 (33.75)	40 (50.00)	**0.030^b^**
	Yes	10 (12.50)	3 (3.75)	

STS, Society of Thoracic Surgeons; TTE, transthoracic echo; TAVI, Transcatheter Aortic Valve Implantation; PCI, a Percutaneous Coronary Intervention; SAPT, Single Antiplatelet Therapy; DAPT, Dual Antiplatelet Therapy; TTE, transthoracic echo; LV eDD, Left Ventricular End-Diastolic Dimension; LVEF, Left Ventricular Ejection Fraction; m, minute; SEV, self-expansile valve; BEV, ballon-expansile valve; mGy·cm^2^, milligray·centimeters squared; AKI, Acute Kidney Injury; MI, myocardial infarction; S/P, Status post.

Bold values indicate statistical significance at *p* < 0.05.

^a^ Independent *t*-test.

^b^ Fisher's exact.

^c^ Two-sample Wilcoxon rank-sum (Mann–Whitney) test.

Table 2 presents the characteristics of patients undergoing Transcatheter Aortic Valve Implantation (TAVI), stratified by the presence of acquired von Willebrand syndrome (AVWS). The mean age for patients without AVWS is 80.36 years, while those with AVWS have a mean age of 78.63 years, with no significant difference (*p* = 0.404). Gender distribution shows a similar percentage of males and females in both groups, and demographic factors such as the STS score, BMI, and medical history conditions, including diabetes and hypertension, do not demonstrate significant differences. Notably, the prevalence of dyslipidemia is significantly higher in patients without AVWS (*p* = 0.031). Laboratory values indicate that hemoglobin levels are lower in the AVWS group (*p* = 0.148), while total cholesterol is higher (*p* = 0.09), but these differences are not statistically significant. Pre- and post-procedural echocardiographic parameters reveal significant differences in aortic valve area (*p* = 0.046) and mean pressure gradient (*p* = 0.025), suggesting that AVWS may impact hemodynamic outcomes. Complications within 30 days post-TAVI indicate a significant difference in arrhythmias (*p* = 0.046) and major vascular complications (*p* = 0.03), with higher incidences in the AVWS group, highlighting the potential risks associated with this condition in the context of TAVI. Overall, while many demographic and clinical characteristics are similar between groups, the findings emphasize the importance of monitoring patients with AVWS for potential complications following TAVI.

**Table 2:** Characteristics of patients and characteristics of trans-catheter aortic valve implantation (TAVI) according to acquired von willebrand syndrome (AVWS).

Variable	Category	No- acquired von Willebrand syndrome (AVWS) (*n* = 36)	Acquired von Willebrand syndrome (AVWS) (*n* = 44)	*P*-value
Demographic data				
Age, mean ± SD, y	Mean ± SD	80.36 ± 6.74	78.63 ± 10.72	0.404^a^
Gender *n*, %	Male	21 (26.25)	22 (27.50)	0.50^b^
	Female	15 (18.75)	22 (27.50)	
STS Score	Mean ± SD	7.11 ± 1.1	6.98 ± 1.42	0.635^a^
BMI, mean ± SD, kg/m^2^	Mean ± SD	28.54 ± 4.81	27.60 ± 5.21	0.41^a^
Medical History				
History Diabetes Mellitus	No	20 (25.00)	25 (31.25)	1.000^b^
	Yes	16 (20.00)	19 (23.75)	
History Hypertension	No	5 (6.25)	6 (7.50)	1.000^b^
	Yes	31 (38.75)	38 (47.50)	
Smoker	No	26 (32.50)	37 (46.25)	0.785^b^
	Yes	10 (12.50)	7 (8.75)	
History Dyslipidemia	No	2 (2.50)	11 (13.75)	**0.031** ^b^
	Yes	34 (42.50)	33 (41.25)	
History Atrial fibrillation	No	24 (30.00)	22 (27.50)	0.822^b^
	Yes	12 (15.00)	22 (27.50)	
History Coronary Artery Disease (CAD)	No	14 (17.50)	18 (22.50)	1.00^b^
	Yes	22 (27.50)	26 (32.50)	
History Peripheral Vascular Disease (PVD)	No	22 (27.50)	33 (41.25)	0.228^b^
	Yes	14 (17.50)	11 (13.75)	
History Myocardial Infarction (S/P MI)	No	29 (36.25)	31 (38.75)	0.437^b^
	Yes	7 (8.75)	13 (16.25)	
History Cerebrovascular Accident—Transient Ischemic Attack	No	33 (41.77)	35 (44.30)	0.328^b^
	Yes	3 (3.80)	8 (10.13)	
Pacemaker	No	27 (33.75)	34 (42.50)	1.00^b^
	Yes	9 (11.25)	10 (12.50)	
S/P CABG	No	34 (42.50)	37 (46.25)	0.175^b^
	Yes	2 (2.50)	7 (8.75)	
S/P TAVI PCI	No	23 (28.75)	26 (32.50)	0.81^b^
	Yes	13 (16.25)	18 (22.50)	
Laboratory				
Hemoglobin (g/dl)	Mean ± SD	12.17 ± 1.59	11.61 ± 1.81	0.148^a^
White blood cells (K/ul)	Mean ± SD	7.54 ± 2.13	7.13 ± 2.42	0.43^a^
Neutrophil-absolute (K/ul)	Mean ± SD	5.1 ± 1.72	4.91 ± 2.42	0.692^a^
Lymphocytes-absolute (K/ul)	Mean ± SD	1.49 ± 0.97	1.63 ± 1.86	0.694^a^
NLR	Mean ± SD	4.55 ± 3.94	4.28 ± 5.13	0.79^a^
Platelets (K/ul)	Mean ± SD	177.41 ± 55.4	181.97 ± 78.29	0.76^a^
Total cholesterol (mg/dl)	Mean ± SD	133.11 ± 21.9	141.77 ± 23.79	0.09^a^
Total protein (g/dl)	Mean ± SD	6.86 ± 0.75	6.79 ± 0.72	0.67^a^
Albumin (g/dl)	Mean ± SD	3.85 ± 0.31	3.77 ± 0.32	0.25^a^
Creatinine (mg/dl)	Mean ± SD	1.41 ± 1.02	1.30 ± 0.65	0.57^a^
Antiplatelets				
SAPT	No	29 (36.25)	40 (50.00)	0.208^b^
	Yes	7 (8.75)	4 (5.00)	
DAPT	No	34 (42.50)	43 (53.75)	0.585^b^
	Yes	2 (2.50)	1 (1.25)	
Anticoagulants	No	30 (37.50)	40 (50.00)	0.333^b^
	Yes	6 (7.50)	4 (5.00)	
Preprocedural TTE				
Septum thickness, millimeters (mm)	Mean ± SD	12.5 ± 1.85	13.36 ± 2.17	0.06^a^
LVEF%	Median (Q1-Q3)	55 (52.5–55)	55 (50–60)	0.547^c^
Aortic Valve Area (cm^2^)	Mean ± SD	0.94 ± 0.5	0.74 ± 0.39	**0.046** ^a^
Valve Type	SEV	22 (27.50)	26 (32.50)	1.00^b^
	BEV	14 (17.95)	18 (23.08)	
Mean PG (mmHg)	Mean ± SD	41.75 ± 13.5	48.06 ± 11.37	**0.025** ^a^
Max PG (mmHg)	Mean ± SD	71.02 ± 21.39	74.52 ± 24.8	0.5^a^
Post-procedural TTE At nadir (3 days post-procedure)				
Septum thickness, millimeters (mm)	Mean ± SD	12.36 ± 11.67	12.63 ± 12.13	0.5^a^
LVEF%	Median (Q1-Q3)	55 (50–55)	55 (49.5–56.25)	0.588^c^
Mean PG (mmHg)	Mean ± SD	11.02 ± 4.2	8.40 ± 4.09	0.006^a^
Max PG (mmHg)	Mean ± SD	11.63 ± 4.89	14.70 ± 10.59	0.11^a^
Laboratory At nadir (3 days post-procedure)				
White blood cells (K/ul)	Mean ± SD	9.52 ± 3.16	8.63 ± 3.01	0.22^a^
Neutrophil-absolute (K/ul)	Mean ± SD	7.39 ± 2.77	6.69 ± 2.96	0.30^a^
Lymphocytes-absolute (K/ul)	Mean ± SD	1.15 ± 0.64	1.08 ± 0.43	0.56^a^
NLR	Mean ± SD	9.71 ± 14.5	7.98 ± 8.23	0.52^a^
Electrocardiography				
QTc Interval (ms)	Mean ± SD	441.87 ± 25.94	451.8 ± 32.34	0.21^a^
RBBB	No	34 (42.50)	39 (48.75)	0.449^b^
	Yes	2 (2.50)	5 (6.25)	
LBBB	No	36 (45.00)	42 (52.50)	0.499^b^
	Yes	0 (0.00)	2 (2.50)	
QRS Duration	Mean ± SD	126.33 ± 31.02	127.7 ± 32.09	0.87
Complications up to 30 days post-TAVI				
AKI	No	30 (37.50)	40 (50.00)	0.33^b^
	Yes	6 (7.5)	4 (5.00)	
MI	No	35 (43.75)	42 (52.50)	1.00^b^
	Yes	1 (1.25)	2 (2.50)	
Major Bleeding	No	36 (45.00)	41 (51.25)	0.248^b^
	Yes	0 (0.00)	3 (3.75)	
Pacemaker	No	31 (38.75)	42 (52.50)	0.23^b^
	Yes	5 (6.25)	2 (2.50)	
Re-admission	No	33 (41.25)	36 (45.00)	0.32^b^
	Yes	3 (3.75)	8 (10.00)	
Arrhythmias	No	30 (37.50)	27 (33.75)	**0.046** ^b^
	Yes	6 (7.50)	17 (21.25)	
Major vascular complications	No	34 (42.50)	33 (41.25)	**0.03** ^b^
	Yes	2 (2.50)	11 (13.75)	

STS, Society of Thoracic Surgeons; SAPT, Single Antiplatelet Therapy; DAPT, Dual Antiplatelet Therapy; NLR, Neutrophil to Lymphocyte Ratio; LBBB, Left Bundle Branch Block; LVEF, Left Ventricular Ejection Fraction; RBBB, Right Bundle Branch; AVA, Block Aortic Valve Area; PG, pressure gradient; TTE, transthoracic echo; LVEF, Left Ventricular Ejection Fraction; m, minute; AKI, Acute Kidney Injury; MI, myocardial infarction; SEV, Self-Expanding Valves; BEV, Balloon-Expandable Valves; mGy·cm^2^, milligray·centimeters squared; S/P, Status post.

Bold values indicate statistical significance at *p* < 0.05.

^a^ Independent *t*-test.

^b^ Fisher's exact.

^c^ Two-sample Wilcoxon rank-sum (Mann–Whitney) test.

**Figure 2:**
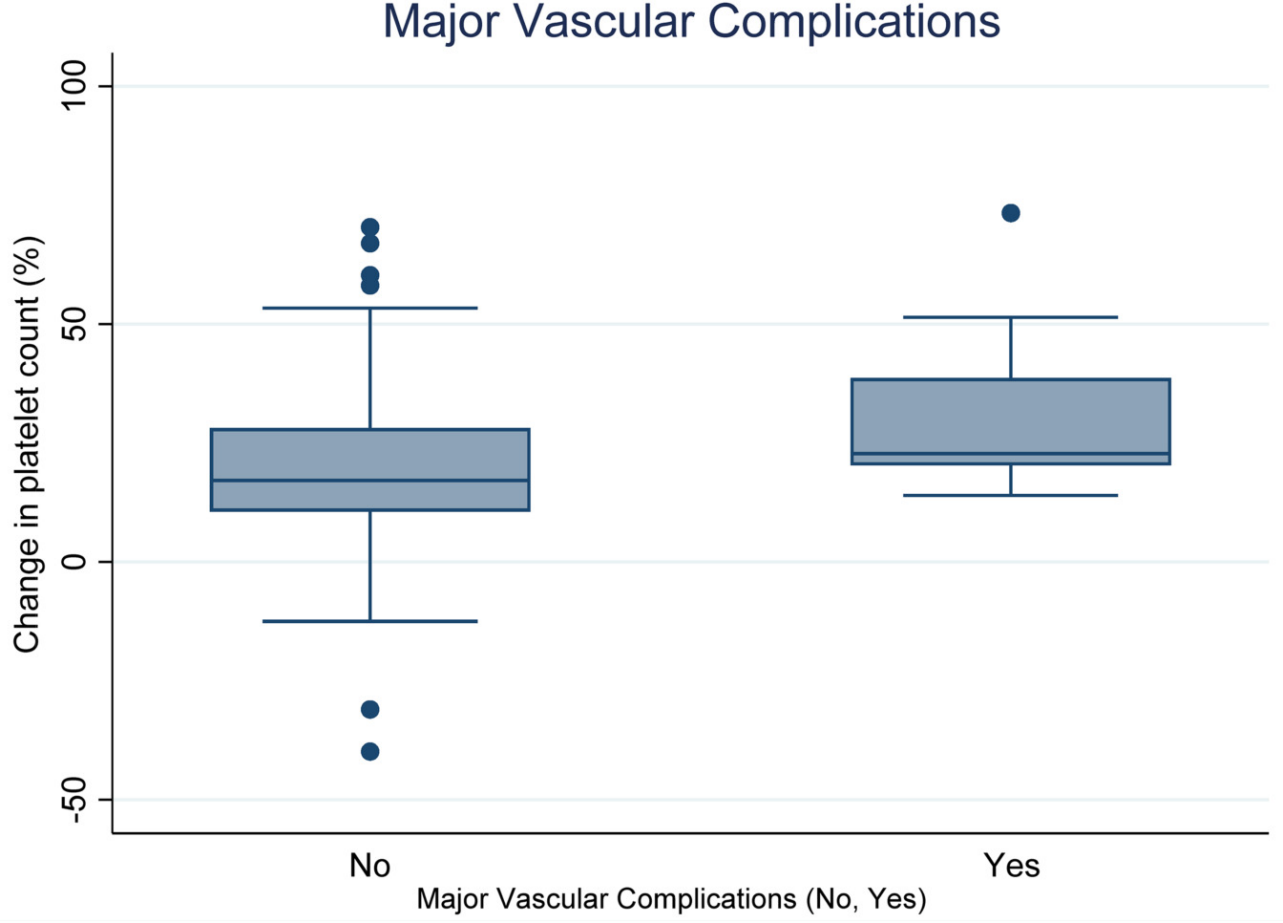
Relationship between decrease in platelet count (DPC) and major vascular complications (Yes vs. No).

Figure 2 present Boxplots display the change in platelet count according to the presence or absence of major vascular complications. The median decreases in platelet count are as follows: for complications present (Yes: 22.75%) and for complications absent (No: 17.1%). Statistical analysis indicates a significant difference between the two groups (*p* = 0.0319).

**Figure 3:**
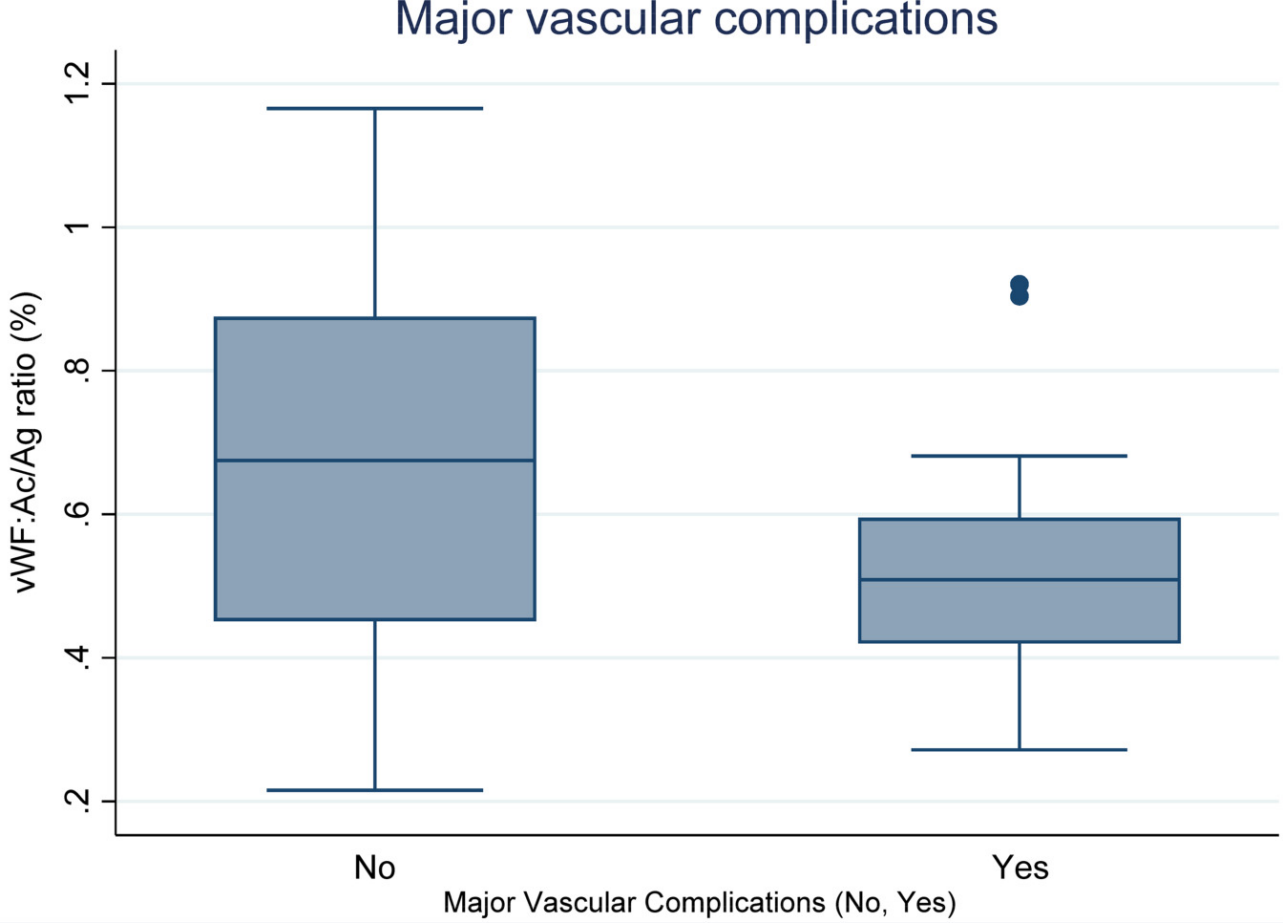
Relationship between vWF: Ac/Ag ratio (%) and major vascular complications (Yes vs. No).

Boxplots Figure 3 illustrates the vWF: Ac/Ag ratio (%) based on the occurrence of major vascular complications. The median changes in platelet count are as follows: for complications present (Yes: 0.50%) and for complications absent (No: 0.74%). Statistical analysis reveals a significant difference between these groups (*p* = 0.0319).

Table 3 presents the results of a multivariable logistic regression analysis evaluating predictors of major vascular complications in a cohort of 80 patients (DPC Model). A decrease in platelet count (DPC) demonstrated a positive association with major vascular complications (coefficient = 1.276, *p* = 0.072), suggesting a potential link, although this result did not reach conventional statistical significance (95% CI: −0.116 to 2.668). None of the other predictors showed statistically significant associations with the outcome. Post-dilatation (coefficient = –0.426, *p* = 0.566), procedural duration (coefficient = 0.010, *p* = 0.340), left ventricular ejection fraction (LVEF) (coefficient = 0.002, *p* = 0.940), septum thickness (coefficient = 0.043, *p* = 0.770), and obesity (coefficient = 0.321, *p* = 0.620). Additionally, the comparison between balloon-expandable valves (BEV) and self-expandable valves (SEV) (coefficient = –0.780, *p* = 0.273) showed no significant difference in the likelihood of vascular complications.

**Table 3:** Predictor variables for Major vascular complications in a multivariable logistic regression analysis: DPC model.

Major vascular complications, (*n* = 80)	Coef	*p*	95% confidence interval
Decrease in Platelet Count (DPC)	1.276	0.072	−0.116, 2.668
Post dilatation^a^	−0.426	0.566	−1.88, 1.03
Procedural duration (ms)	0.010	0.34	−0.010, 0.031
LVEF^a^	0.002	0.94	−0.068, 0.074
Septum thickness^a^	0.043	0.77	−0.245, 0.332
Obesity^a^	0.321	0.62	−0.946, 1.588
BEV vs. SEV	−0.780	0.273	−2.175, 0.614

^a^ Baseline.

LVEF, Left Ventricular Ejection Fraction; SEV, Self-Expandable Valves; BEV, Balloon-Expandable Valves; ms, minutes.

The variance inflation factor (VIF) values reveal that multicollinearity is not a concern, with all values below 2. The mean VIF is 1.14, indicating that predictor variables are not highly correlated. In the generalized additive model analysis, the total gain from the nonlinearity chi-square test is 6.635 (df = 8.383) with a *p*-value of 0.6157, indicating that the model does not significantly capture nonlinear relationships between the predictors and the outcome.

In effect modification analysis, none of the interaction terms between DPC and the other predictor variables were statistically significant, suggesting that the relationship between DPC and major vascular complications is consistent across different levels of the other predictors.

Table 4 summarizes the results of the multivariable logistic regression analysis assessing predictors of major vascular complications in 80 patients (AVWS Model). Acquired von Willebrand syndrome (AVWS) was identified as a statistically significant predictor (coefficient = 1.841, *p* = 0.022; 95% CI: 0.271–3.410), indicating a strong association with increased risk.

**Table 4:** Predictor variables for major vascular complications in a multivariable logistic regression analysis: acquired von Willebrand syndrome (AVWS) model.

Major vascular complications, (*n* = 80)	Coef	*p*	95% confidence interval
acquired von Willebrand syndrome (AVWS)	1.841	0.022	0.271, 3.410
Post dilatation^a^	0.101	0.895	−1.401, 1.604
Procedural duration (ms)	0.015	0.175	−0.006, 0.037
LVEF^a^	0.013	0.697	−0.055, 0.083
Septum thickness^a^	−0.142	0.381	−0.461, 0.176
Obesity^a^	0.846	0.209	−0.474, 2.168
BEV vs. SEV	−1.076	0.16	−2.576, 0.424

^a^ Baseline.

LVEF, Left Ventricular Ejection Fraction; SEV, Self-Expanding Valves; BEV, Balloon-Expandable Valves; ms, minutes.

None of the other covariates showed statistically significant associations. Post-dilatation (Coef = 0.101, *p* = 0.895), procedural duration (Coef = 0.015, *p* = 0.175), LVEF (Coef = 0.013, *p* = 0.697), septum thickness (Coef = –0.142, *p* = 0.381), and obesity (Coef = 0.846, *p* = 0.209) all had confidence intervals crossing zero, indicating no clear effect. The comparison between balloon-expandable valves (BEV) and self-expanding valves (SEV) (Coef = –1.076, *p* = 0.160) also showed a non-significant trend toward lower risk with BEV, but this did not reach statistical significance.

The variance inflation factors (VIFs) for the predictor variables indicated low levels of multicollinearity, with all VIF values below 2.0, and a mean VIF of 1.14. This suggests that multicollinearity is not a concern in the regression analysis.

A Generalized Additive Model was employed to explore potential nonlinear relationships between the continuous predictors and major vascular complications. The total gain from the nonlinearity chi-square test was 6.645 with 8.183 degrees of freedom, yielding a *p*-value of 0.7157, indicating no significant evidence of nonlinearity among the continuous predictors.

In effect modification analysis, none of the interaction terms between AVWS and the other predictor variables were statistically significant, suggesting that the relationship between AVWS and major vascular complications is consistent across different levels of the other predictors.

## AVWS model and DPC model comparison

### AIC and BIC for fitting data

To compare the predictive performance of the two models, we fitted multivariable logistic regression models using either DPC or AVWS as the primary predictor, along with shared covariates. The AVWS model demonstrated superior fit, as evidenced by a AIC of 74.33 and BIC of 93.38, compared to the DPC model (AIC = 78.47; BIC = 97.53). Additionally, the AVWS model yielded a higher pseudo-R^2^ value (0.1786 vs. 0.1202) and a more favorable likelihood ratio chi-square statistic (*χ*^2^ = 12.68, *p* = 0.080) than the DPC model (*χ*^2^ = 8.53, *p* = 0.288). These results suggest that AVWS may be a more robust and discriminative predictor of major vascular complications following TAVI, offering potential value in pre-procedural risk stratification.

**Figure 4:**
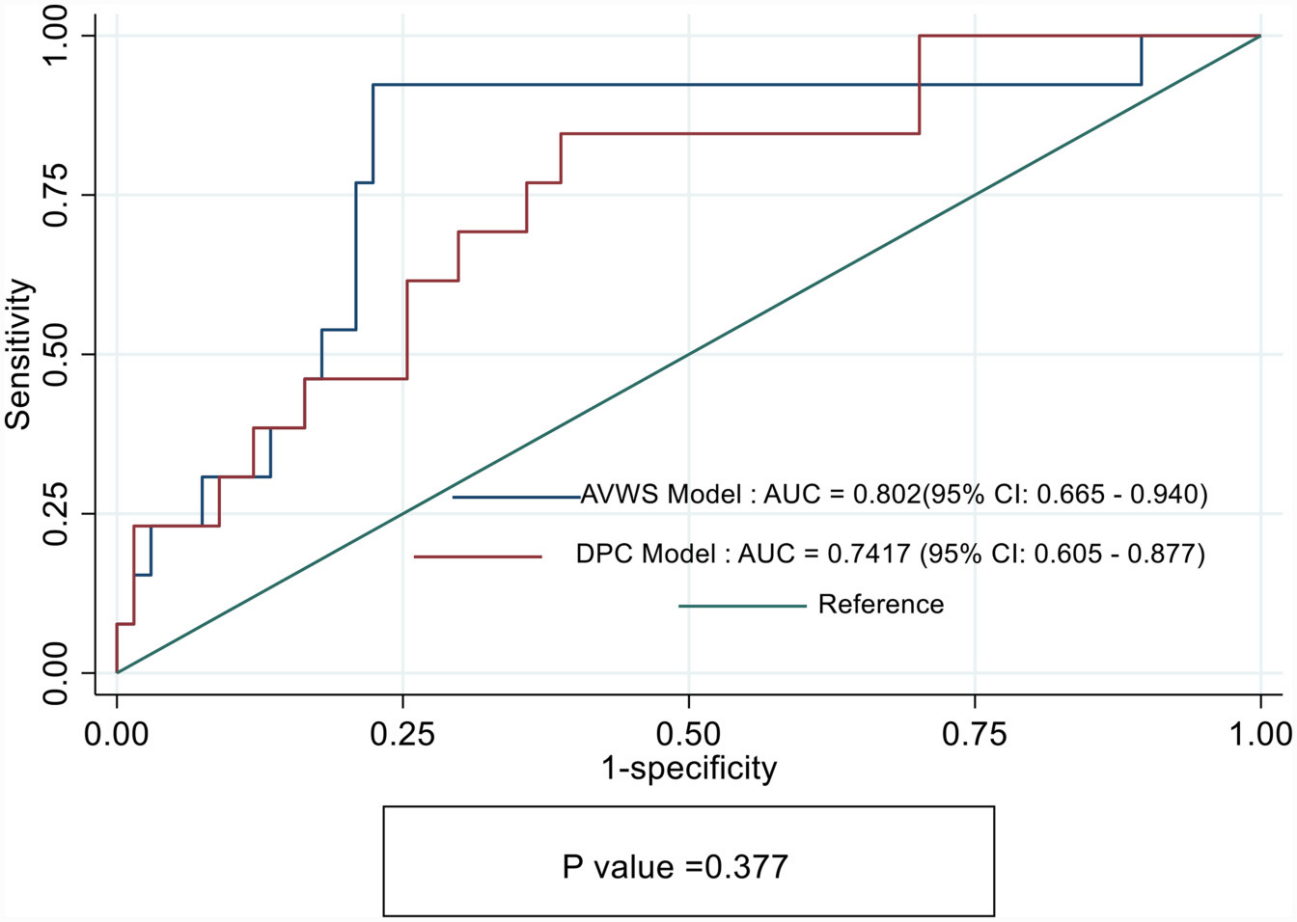
Receiver operating characteristics (ROC) curves showing sensitivity and specify of DPC model and acquired von Willebrand syndrome (AVWS) model for prediction of Major vascular complications in TAVI cohorts.

Figure 4 shows that the AUC for the AVWS Model was significantly higher than that of the DPC Model, suggesting better discriminative ability. However, comparing the AUCs between the two models yielded a chi-square statistic of 0.78 with a *p*-value of 0.377, indicating no statistically significant difference in performance.

Table 5 shows that the most common arrhythmias after TAVI were atrial fibrillation and new LBBB (21.7% each), followed by complete AVB (17.4%). Other conduction disturbances, including first- and second-degree AVB, were also observed.

**Table 5:** Types of arrhythmias after TAVI.

Type of arrhythmias	Frequency	Precent
First degree AVB	3	13.0%
Second degree AVB Mobitz Type I	2	8.7%
Second degree AVB Mobitz Type II	1	4.3%
Complete AVB	4	17.4%
New LBBB	5	21.7%
HV Interval >70 ms	2	8.7%
Atrial fibrillation	5	21.7%
Ventricular tachycardia	1	4.3%

Atrioventricular Block (AVB) data present in count (%).

Table 6 presents the results of the multivariable logistic regression analysis conducted to identify predictors of arrhythmias among 80 patients. Acquired von Willebrand syndrome (AVWS) was found to be a significant predictor, with an odds ratio of 4.48 (*p* = 0.024), suggesting that patients with AVWS have more than four times the odds of experiencing arrhythmias compared to those without the condition [95% CI: (1.22, 16.49)]. Other nonsignificant predictors included a decrease in platelet count (DPC), with an odds ratio of 0.36 [*p* = 0.167; 95% CI: (0.09, 1.53)], and post-dilatation [OR = 0.90, *p* = 0.892; 95% CI: (0.19, 4.18)], which showed no meaningful effect on arrhythmia risk.

**Table 6:** Predictor variables for arrhythmias in a multivariable logistic regression analysis.

Arrhythmias, (*n* = 80)	Odds ratio	*p*	95% confidence interval
AVWS	4.480369	0.024	1.217166–16.49217
DPC	0.364537	0.167	0.0870543–1.526489
Post dilatation^a^	0.898645	0.892	0.1930743–4.182657
Procedural duration (m)	1.031352	0.008	1.008147–1.055091
Thrombocytopenia^b^	2.472231	0.186	0.6463273–9.456396
LVEF^a^	1.050393	0.179	0.9777369–1.128448
Septum thickness^a^	0.965888	0.805	0.7327622–1.273181
Obesity^a^	1.85008	0.333	0.5321935–6.431485
BEV vs. SEV	0.441981	0.209	0.12371–1.579076

^a^ Baseline.

^b^ At nadir (3 days post-procedure);.

LVEF, Left Ventricular Ejection Fraction; AVWS, acquired von Willebrand syndrome.

The procedural duration was positively associated with arrhythmias, with an odds ratio of 1.03 [*p* = 0.008; 95% CI: (1.01, 1.06)], indicating that longer procedural durations may increase the likelihood of arrhythmias. Thrombocytopenia showed an odds ratio of 2.47 [*p* = 0.186; 95% CI: (0.65, 9.46)], reflecting a potential but statistically **insignificant association**.

The LVEF exhibited an odds ratio of 1.05 [*p* = 0.179; 95% CI: (0.98, 1.13)], indicating a slight increase in risk, while septum thickness exhibited an odd ratio of 0.097 [*p* = 0.805; 95% CI: (0.73, 1.27)], both were not significantly associated with arrhythmias as their *p* values were more than 0.05. Obesity demonstrated an odds ratio of 1.85 [*p* = 0.333; 95% CI: (0.53, 6.43)], and BEV vs. SEV showed an odds ratio of 0.44 [*p* = 0.209; 95% CI: (0.12, 1.58)], both indicating no significant associations with arrhythmia risk.

The goodness-of-fit test using the Hosmer–Lemeshow statistic indicated no significant lack of fit, with a chi-square value of 8.57 and a *p*-value of 0.3795.

The analysis of multicollinearity revealed low levels, with a mean-variance inflation factor (VIF) of 1.25, suggesting that multicollinearity was not a concern in this model.

the Generalized Additive Model (GAM) analysis demonstrated no significant evidence of nonlinearity among the continuous predictors, as reflected in the total gain chi-square of 4.687 (*p* = 0.8355).

**Figure 5:**
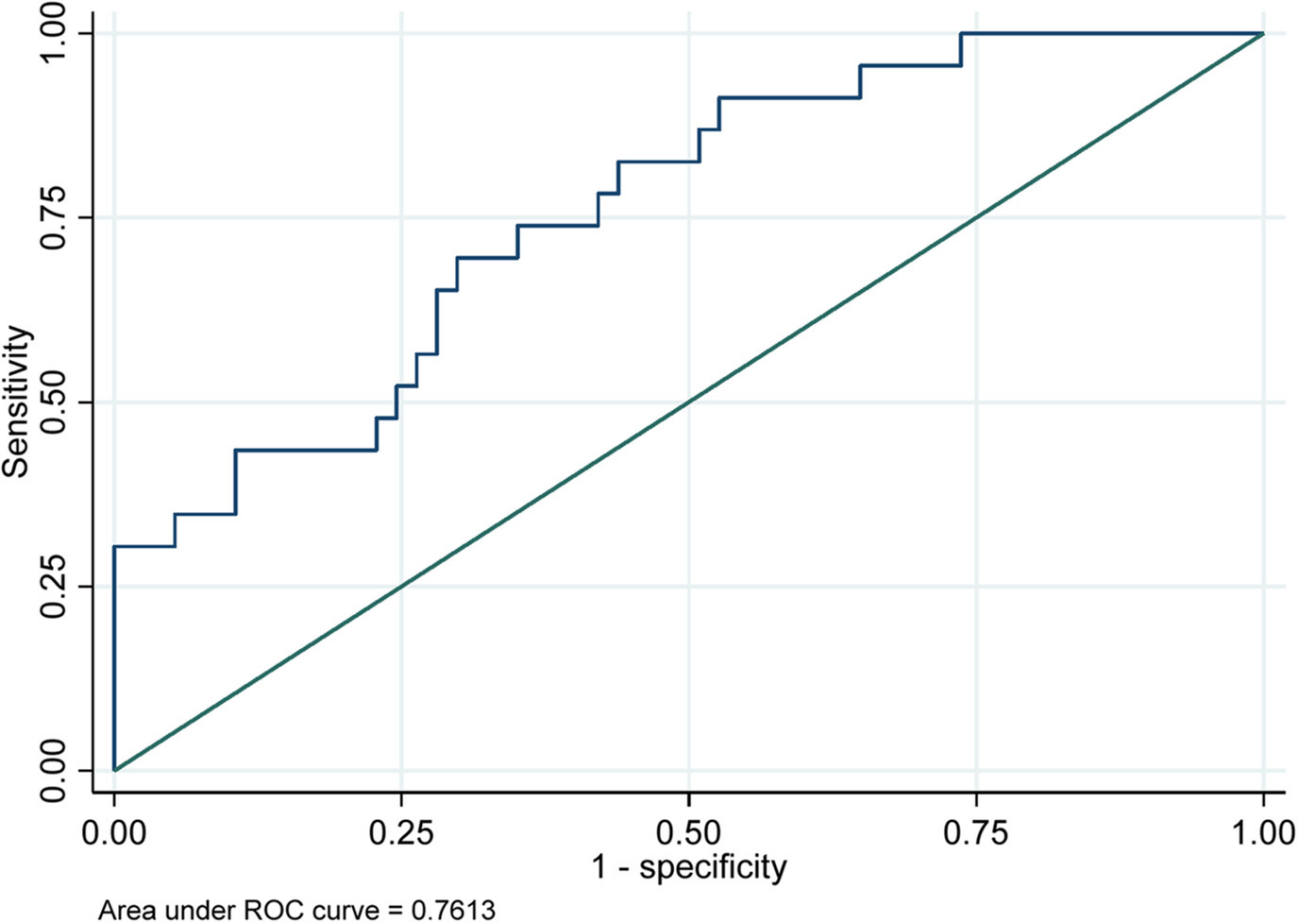
Receiver operating characteristics (ROC) curves for prediction of arrhythmia complications in TAVI cohorts.

Figure 5 presents the model demonstrated good discriminative ability, with an area under the receiver operating characteristic (ROC) curve of 0.7613. This indicates that the model effectively differentiates between patients who develop arrhythmias and those who do not, suggesting that the predictors included in the model are relevant for understanding arrhythmia risk in the studied population.

## Discussion

This study aimed to explore the predictive factors for major vascular complications and arrhythmias following Transcatheter Aortic Valve Implantation (TAVI), focusing on the roles of decrease in platelet count (DPC) and acquired von Willebrand syndrome (AVWS). We developed two separate predictive models—one based on DPC and the other on AVWS—for predicting major vascular complications as the primary outcome. To the best of our knowledge, this is the first direct comparison of these two models in the context of TAVI. Additionally, we developed a separate model to predict arrhythmias following TAVI, our findings indicate that AVWS is a superior predictor compared to DPC in identifying patients at risk for both major vascular complications and arrhythmias following TAVI. These findings highlight the potential clinical utility of AVWS in stratifying patient risk for both vascular and arrhythmic complications after TAVI.

### Implications of decrease in platelet count

Previous studies demonstrated that TAVI can induce temporary thrombocytopenia (25). Several factors contribute to a DPC following TAVI. The precise reasons for DPC remain unclear (26–28). This study analyzed several of these factors, as outlined in Table 1. We observed a significant drop in platelet count following TAVI, this finding corresponds to the observations in Nastasia Roth's study, further substantiating the prevalence of DPC in this patient population (17).

In previous studies, DPC has been linked to adverse outcomes in various cardiac procedures, including TAVI (29). Our study confirmed that significant decreases in platelet count correlate with an increased risk of major vascular complications. This aligns with existing literature suggesting that thrombocytopenia can compromise hemostatic mechanisms, leading to heightened vulnerability during and after the procedure (4, 7, 8). However, the lack of statistical significance in some of our analyses indicates that DPC alone may not fully capture the complexity of the coagulation changes occurring in this patient population.

### Role of acquired von Willebrand syndrome

Previous studies the prevalence of abnormal vWF multimers in AS patients varies among studies, ranging from 20 to 70% (30, 31). However, our study revealed a prevalence of 55%. Screening for vWF abnormalities can be inconsistent due to the presence of multiple assays, including vWF-ristocetin cofactor activity (vWF: RCo), vWF collagen binding (vWF: CB), CT-ADP membrane closure time with the Platelet Function Analyzer (PFA), and gel electrophoresis (32). However, there are significant variations across electrophoretic studies, likely because of varying AS severity among groups (33). The vWF: Ac assay appears to be less affected by high bilirubin, free hemoglobin, lipidemia, or genetic polymorphism than the vWF: RCo assay, allowing for more reliable screening of AVWS, particularly in the setting of cardiac valve disease and mechanical circulatory support (34, 35).

The importance of AVWS from a retrospective cohort study conducted before intervention lies in its association with an increased risk of major bleeding complications (11). This finding is supported by our study, which demonstrated that AVWS exhibits strong predictive ability in multivariate logistic regression, even after adjusting for all confounding factors. This is particularly important, as AVWS can lead to impaired platelet function and increased bleeding risk, which may contribute to the observed complications.

In our study, we identified a significant relationship between Acquired von Willebrand Syndrome (AVWS) prior to Transcatheter Aortic Valve Implantation (TAVI) and the incidence of arrhythmias post-procedure. We attribute this association to several key mechanisms: AVWS disrupts hemostatic balance, influences inflammatory responses, and impairs endothelial function—all of which are essential for maintaining normal cardiac rhythm (4, 10, 19, 31).

The findings suggest that screening for AVWS before TAVI could enhance risk stratification and inform clinical decision-making.

### Comparative predictive value of AVWS and platelet count decrease in major vascular complications

This study aimed to evaluate and compare the predictive performance of two hemostatic biomarkers—AVWS and DPC—in forecasting major vascular complications following TAVI. While both markers demonstrated mechanistic plausibility, AVWS emerged as a statistically significant predictor in multivariable analysis (*p* = 0.022), whereas DPC did not reach the threshold for statistical significance (*p* = 0.072). Additionally, the AVWS model exhibited a better overall fit, reflected by lower AIC and BIC values and a higher pseudo-R^2^, indicating enhanced explanatory power. These findings align with prior evidence linking AVWS to vascular injury and bleeding risk in high-shear interventional settings (11).

Although the AVWS model achieved a higher area under the ROC curve (AUC = 0.80) compared to the DPC model (AUC = 0.74), the difference between the two models was not statistically significant (*p* = 0.377). We interpret this finding with appropriate caution, acknowledging that both models performed comparably in terms of overall discrimination. However, AUC alone may not fully capture the predictive value of a biomarker—particularly when one variable, such as AVWS, demonstrates statistical significance in multivariable analysis, stronger model diagnostics (lower AIC/BIC, higher pseudo-R^2^), and clear biological plausibility. The consistent statistical and clinical performance of AVWS, combined with its mechanistic link to shear-induced vascular injury, supports its potential as a more reliable and clinically actionable biomarker for pre-procedural risk stratification in TAVI patients.

From a clinical perspective, incorporating AVWS assessment into pre-TAVI evaluations could allow for more personalized decision-making, including vascular access planning and perioperative bleeding risk management. While DPC remains an important indicator of procedural stress and hemostatic consumption, AVWS may offer a more upstream and mechanistically targeted view of vascular vulnerability. These findings should be regarded as hypothesis-generating, but they lay the groundwork for larger-scale validation studies that could solidify the role of AVWS in improving patient outcomes in structural heart interventions.

### Acquired von Willebrand syndrome and procedural duration as predictors of arrhythmias

AVWS significantly impacts the risks associated with cardiac arrhythmias, primarily due to its effect on endothelial function, inflammation, and hemostatic balance. The relationship between AVWS and arrhythmias stems from several interrelated mechanisms that disrupt normal cardiac electrophysiology and structural integrity.

AVWS disrupts the balance of hemostasis, primarily by diminishing the functional levels of vWF. This deficiency can result in a hypercoagulable state, leading to abnormal clot formation and potential vessel occlusion, which can in turn result in ischemia and subsequent arrhythmias (36, 37). The relationship between endothelial function and health in patients with AVWS is crucial; endothelial dysfunction, often present in AVWS patients, is linked to increased inflammation and impaired vasodilation (38, 39). Poor endothelial health contributes directly to arrhythmias, particularly atrial fibrillation (AF), by promoting fibrosis and structural changes within the heart muscle, disrupting normal electrical conduction (40, 41).

Moreover, inflammation plays a critical role in the pathology observed in AVWS. Elevated inflammatory markers have been associated with worsening vascular integrity and a heightened risk of arrhythmias (42, 43). This connection is further highlighted by the potential for inflammatory mediators to exacerbate endothelial dysfunction, demonstrating a dual pathway through which AVWS may increase arrhythmia risk: by direct impairment of vascular function and by promoting an inflammatory milieu that disrupts cardiac rhythm (39, 41).

The evidence suggests that advanced metrics of endothelial function and inflammatory status should be integrated into pre-procedural assessments for patients with AVWS undergoing catheter-based interventions. This would facilitate stratified risk management and help in clinical decision-making regarding arrhythmia prevention strategies during and after procedures like TAVI. Furthermore, targeting the underlying inflammation and improving endothelial function through therapeutic interventions may serve as avenues for reducing the incidence of arrhythmias in this vulnerable population.

According to Table 6, The analysis identified acquired von Willebrand syndrome (AVWS) and procedural duration as significant and robust predictors of arrhythmias. Patients with AVWS demonstrated over four times the odds of developing arrhythmias compared to those without the condition, while longer procedural durations were associated with an increased risk, emphasizing procedural duration as a critical factor. Although post-dilatation may exacerbate mechanical stress on the interventricular septum, potentially leading to new-onset arrhythmias (44, 45), its *p*-value was not significant. Similarly, while valve heterogeneity could influence the study's strength, valve type (BEV vs. SEV) was also nonsignificant. These findings highlight that, even after adjusting for and controlling these variables, AVWS and procedural duration remain the most significant and independent predictors of arrhythmia risk.

### Risk of overfitting and model validation strategies

Prior research has emphasized the significant role of metabolic syndrome components—particularly obesity in influencing post- TAVI complications. this comorbidity has been associated with adverse outcomes such as vascular injury, delayed recovery, endothelial dysfunction, and increased inflammation, all of which can affect recovery trajectories and overall patient prognosis after the procedure (46–48). The relationship between these conditions and post-TAVI events underscores the pivotal impact of metabolic health on procedural outcomes in patients undergoing TAVI.

Recent studies demonstrate that the presence of metabolic syndrome is linked to a heightened risk of complications such as major bleeding, conditions that exacerbate the burden of cardiovascular morbidity in this population (48). Notably, evidence shows that bleeding risks are prominently associated with procedural complications, which can result from access-related issues that are frequent during TAVI procedures. For instance, the presence of obesity may amplify these risks due to its pro-inflammatory effects and potential relation with thromboembolic events.

The inclusion of variables such as obesity in our multivariable models, despite their lack of statistical significance, is a deliberate decision rooted in established literature recognizing these factors as significant contributors to cardiovascular morbidity. Obesity has been consistently linked to an increased cardiovascular risk, which can adversely affect clinical outcomes TAVI (23, 24, 49, 50). Previous studies highlight that obesity and its associated conditions often exacerbate cardiovascular events and complications, underscoring the necessity of incorporating such variables into predictive models (22, 50, 51).

Moreover, excluding these variables may lead to residual confounding, particularly in observational studies where many known risk factors influence the outcomes being studied. This is supported by systematic evidence indicating that not accounting for obesity-related cardiovascular risks could skew results, potentially leading to an underestimation of the predictive accuracy of our models. However, we recognize that the number of predictors relative to the number of outcome events is a critical concern, particularly when the EPV ratio is below the conventional threshold of 10. A low EPV can destabilize model estimates and lead to overfitting, which may inflate predictive performance.

To address this methodological challenge, we utilized Firth's penalized likelihood regression, which is particularly well-suited for small samples and low event rates, as in our dataset. Firth regression applies a bias-reduction method that corrects the small-sample bias commonly encountered in maximum likelihood estimation. This approach provides a robust mechanism to evaluate and adjust for potential overfitting, producing more stable and accurate parameter estimates. Unlike conventional logistic regression, which may yield inflated odds ratios or fail to converge in the presence of sparse data or quasi-complete separation, Firth regression ensures finite and interpretable estimates. This method has been widely recognized in predictive modeling for enhancing the credibility of findings by reducing both bias and variance in effect estimates. In our study, the application of Firth regression contributed to a more reliable assessment of the associations between clinical predictors—such as DPC and obesity—and the occurrence of major vascular complications post-TAVI.

### Limitations

While our study offers important insights, several limitations should be acknowledged. First, the relatively small sample size, while adequate for exploratory analysis, may limit the statistical power and generalizability of the findings. Second, the observational nature of the study precludes any definitive conclusions about causality between the investigated predictors—such as AVWS and DPC—and clinical outcomes following TAVI. Third, the lack of external validation cohorts restricts the broader applicability of our predictive models. Future studies involving larger, multicenter populations are needed to confirm our results and assess the clinical utility of von Willebrand Factor–based risk stratification. Finally, due to post-estimation limitations of the firthlogit command in Stata, we were unable to directly extract predicted probabilities from the Firth models. Consequently, ROC curves and AUC values were derived using standard logistic regression models with the same covariates. While this approach does not incorporate Firth's penalized likelihood correction, it offers an approximate measure of model discrimination for comparative purposes.

### Future directions and conclusion

Further research is needed to investigate the long-term implications of DPC and AVWS on patient outcomes following TAVI. Longitudinal studies could provide insights into how these factors influence recovery and complications over time. Additionally, exploring potential therapeutic interventions aimed at correcting abnormal von Willebrand Factor function could be a promising area for future investigation.

In conclusion, our study highlights the critical roles of both DPC and AVWS in predicting major vascular complications and arrhythmias in patients undergoing TAVI. By enhancing our understanding of these predictors, we can improve risk assessment and ultimately enhance the care of patients undergoing this increasingly common procedure.

## Data Availability

The raw data supporting the conclusions of this article will be made available by the authors, without undue reservation.
